# Fetal and neonatal outcomes of pregnancies complicated by maternal sleep-disordered breathing: a systematic review

**DOI:** 10.3389/fphys.2026.1775697

**Published:** 2026-10-05

**Authors:** Mayu Niisaka, Jun Hosomichi, Sakina Mitsuishi, Shiho Kino, Takashi Ono

**Affiliations:** 1 Department of Orthodontic Science, Graduate School of Medical and Dental Sciences, Institute of Science Tokyo, Tokyo, Japan; 2 Department of Preventive Oral Health Care Sciences, Graduate School of Medical and Dental Sciences, Institute of Science Tokyo, Tokyo, Japan; 3 Center of Excellence in Precision Medicine and Digital Health, Department of Physiology, Faculty of Dentistry, Chulalongkorn University, Bangkok, Thailand

**Keywords:** birth outcome, fetal growth, obstructive sleep apnea, pregnancy, sleep-disordered breathing

## Abstract

**Introduction:**

Maternal obstructive sleep apnea (OSA) and the broader spectrum of sleep-disordered breathing (SDB) during pregnancy may be associated with fetal and neonatal outcomes, but the literature is heterogeneous and uses clinically distinct exposure definitions. This systematic review aimed to evaluate associations between maternal SDB and fetal/neonatal outcomes while preserving differences in exposure ascertainment.

**Methods:**

Following PRISMA 2020, the review was re-audited using updated PubMed and Web of Science searches, backward and forward citation searching, and ClinicalTrials.gov and WHO ICTRP registry searches. Exposures were classified as (1) objectively confirmed OSA/SDB, (2) administrative-code-defined OSA, and (3) symptom-, snoring-, or questionnaire-defined SDB risk. No meta-analysis was conducted because clinically comparable subsets were insufficient for quantitative pooling.

**Results:**

The completed re-audit identified 64 eligible publications mapped to 53 conservative cohort/dataset families (55 if two probable overlap pairs are treated as separate): 22 objective/physiologic, 11 administrative/clinical, and 31 symptom/questionnaire publications. Objective studies showed mixed findings, with fetal-growth or neonatal signals in selected cohorts but null findings in several other objectively assessed populations. Administrative-code OSA was repeatedly associated with preterm birth and selected neonatal outcomes, although exposure misclassification and residual confounding remain important. Symptom/questionnaire studies were heterogeneous, with associations varying by symptom definition, timing, and outcome.

**Conclusion:**

Current evidence does not establish a consistent fetal or neonatal risk pattern for objectively confirmed maternal OSA/SDB. Administrative-code and symptom/questionnaire evidence suggests associations for selected outcomes, particularly preterm birth, but should not be interpreted as a single homogeneous OSA effect.

**Systematic review registration:**

https://www.crd.york.ac.uk/PROSPERO/, identifier CRD420251110706.

## 1 Introduction

Maternal obstructive sleep apnea (OSA), a clinically defined form of sleep-disordered breathing (SDB), is characterized by recurrent upper-airway obstruction with intermittent hypoxemia and sleep fragmentation. Physiological changes during gestation, including weight gain, upper-airway edema, and altered respiratory mechanics, may worsen pre-existing OSA or contribute to new-onset disease. Because snoring, questionnaire positivity, administrative diagnosis codes, and objectively measured OSA/SDB capture different constructs, these exposure definitions should not be treated as interchangeable.

Prior observational studies and systematic reviews have examined maternal SDB in relation to hypertensive disorders, gestational diabetes, birth weight, fetal growth, preterm birth, Apgar scores, and other perinatal outcomes, but findings have varied by exposure definition, population, outcome definition, and covariate adjustment (Pamidi et al., 2014; Ding et al., 2014; Brown et al., 2018; Li et al., 2018; Liu et al., 2019; Sanapo et al., 2024). Therefore, the unresolved question is not whether the topic has previously been reviewed, but how the fetal and neonatal evidence differs when studies are separated by exposure ascertainment and interpreted in light of study-level confounding and risk of bias.

The Developmental Origins of Health and Disease framework provides a biological context for evaluating these associations. Intermittent maternal hypoxemia, oxidative stress, inflammation, metabolic dysregulation, and altered placental perfusion are plausible pathways through which maternal SDB could influence fetal development. However, biological plausibility does not establish a causal association, and mechanistic evidence should be interpreted separately from the observational fetal and neonatal evidence. Related reports have also addressed questionnaire-based OSA risk, maternal adverse outcomes, inflammatory or metabolic pathways, and infant neurodevelopment (Fernández Alonso et al., 2015; Lui et al., 2021; Serednytskyy et al., 2022; Tauman et al., 2015).

Accordingly, this systematic review aimed to update and critically evaluate observational evidence on fetal and neonatal outcomes in pregnancies with maternal SDB. The revised synthesis separates objectively confirmed OSA/SDB, administrative-code-defined OSA, and symptom/snoring/questionnaire-defined SDB risk; examines exposure timing and severity where reported; and emphasizes study-level effect estimates, confidence intervals, confounder adjustment, and risk-of-bias considerations rather than statistical-significance counting alone. The review focuses on predefined outcomes including birth anthropometrics, FGR/IUGR, SGA/LGA, gestational age and preterm birth, Apgar scores, and neonatal adiposity/body composition.

## 2 Methods

### 2.1 Literature search

This systematic review was conducted according to the PRISMA 2020 statement (Page et al., 2021). The original database search included PubMed and Web of Science from database inception to August 30, 2026. The registered PubMed strategy combined three concept blocks—maternal OSA/SDB, pregnancy, and a fetal-growth/outcome block—and retrieved 141 records. The original exposure block included OSA and SDB terms but did not include a specific snoring term, while the outcome block focused on fetal growth, birth weight, birth length, offspring, adiposity, and the Fetal Development MeSH term. The PROSPERO record (CRD420251110706) states a review start date of August 25, 2025, first submission on September 12, 2025, and registration/publication of Version 1.0 on September 17, 2025; Version 1.1 was published on October 21, 2025 and recorded progress in the review. Accordingly, the review is not described as prospectively registered. Version 1.0 already prespecified birth weight, birth length, FGR/IUGR, SGA/LGA, gestational age/preterm birth, neonatal adiposity, placental abnormalities, and delivery mode. Apgar scores and NICU/neonatal morbidity were not individually enumerated in the registered outcome list but were considered within the intended broader fetal/perinatal outcome scope and are specified explicitly in this review. The public PROSPERO record did not explicitly exclude questionnaire-defined SDB. For the present review, the operational exposure framework was clarified as three distinct strata: objectively confirmed OSA/SDB, administrative-code-defined OSA, and symptom/snoring/questionnaire-defined SDB risk. This clarification formalized how heterogeneous exposure constructs already represented in the review would be classified and interpreted; it was not an *a priori* three-category protocol feature and was not based on the direction or statistical significance of the study results. The present review retains this framework and applies it consistently to the reconstructed evidence set. Protocol amendments, clarifications, and deviations are summarized in **Supplementary Table 5**.

We conducted an updated, comprehensive search using higher-sensitivity two-concept strategies (maternal OSA/SDB AND pregnancy) without a required outcome block. PubMed was rerun on August 24, 2026 and retrieved 1,205 records; Web of Science Core Collection was rerun on August 28, 2026 and retrieved 1,671 records. Exact cross-database deduplication using PMID or normalized DOI identified 937 Web of Science records already present in PubMed, yielding 1,939 unique database records. Reference lists of included studies and relevant systematic reviews were checked, and forward citation searching was completed on August 29, 2026 using all 64 included primary publications plus three relevant systematic-review seeds. Forward searching yielded 3,093 raw seed-record occurrences and 628 globally unique additional records after deduplication; 626 were excluded at title/abstract screening and two at full-text assessment, with no additional eligible publication identified. ClinicalTrials.gov and the WHO ICTRP were searched on August 30, 2026, yielding 176 and 69 records, respectively; after removal of 51 duplicate NCT registrations across registries, 194 unique registry records remained, and no additional eligible published full report was identified. Embase was considered but was not searched because institutional access was unavailable. Complete database and registry strategies, platforms, search dates, limits, yields, and supplementary-search details are provided in **Supplementary Table 1**.

### 2.2 Eligibility criteria

Eligibility was defined using a Population–Exposure–Comparator–Outcome (PECO) framework, with study design specified separately. Population: pregnant individuals at any gestational age. Exposure: maternal SDB classified for synthesis as (1) objectively confirmed OSA/SDB assessed by polysomnography, home sleep apnea testing, respiratory polygraphy, nocturnal oximetry, WatchPAT, or an equivalent objective respiratory method; (2) administrative-code-defined or clinically recorded OSA; or (3) symptom-, snoring-, or questionnaire-defined SDB risk. Comparator: pregnant individuals without the corresponding exposure. Outcomes: at least one predefined fetal or neonatal outcome, including birth weight or length, FGR/IUGR, SGA/LGA, gestational age at delivery, preterm birth, Apgar scores at 1 or 5 minutes, or neonatal adiposity/body composition. Eligible designs were observational cohort, case–control, or cross-sectional studies reported in English.

Reviews, meta-analyses, editorials, commentaries, book chapters, conference abstracts, animal or *in vitro* studies, case reports, studies without an appropriate comparator, and reports without a predefined fetal or neonatal outcome were excluded from the primary evidence set. Screening or symptom positivity was not interpreted as a diagnosis of OSA. Delivery mode was not treated as a fetal or neonatal outcome. When multiple publications arose from the same cohort or dataset, they were linked as a report family and were not treated as independent studies for the same outcome.

### 2.3 Study selection

The updated searches identified 2,876 bibliographic database records (PubMed, n=1,205; Web of Science Core Collection, n=1,671). After removal of 937 exact cross-database duplicates by PMID or normalized DOI, 1,939 unique database records were screened. Backward citation reconciliation yielded no unique additional record, whereas forward citation searching across all 64 included primary-publication seeds plus three systematic-review seeds yielded 3,093 raw seed-record occurrences and 628 globally unique additional records after database and cross-seed deduplication. In total, 2,567 publication records were screened and 2,496 were excluded at title/abstract screening. Seventy-one reports were sought and retrieved for full-text assessment; seven were excluded after full-text review (wrong population/comparator, n=1; exposure outside the prespecified OSA/SDB categories, n=3; non-isolatable/broad exposure, n=1; and no eligible exposed-versus-unexposed fetal/neonatal comparison, n=2), leaving 64 eligible publications. These publications mapped to 53 conservative cohort/dataset families; the sensitivity count was 55 if two probable overlap pairs were treated as independent. Registry searching identified 194 unique registrations after cross-register deduplication and yielded no additional eligible published full report. The study selection process is shown in **Figure 1**.

**Figure 1:**
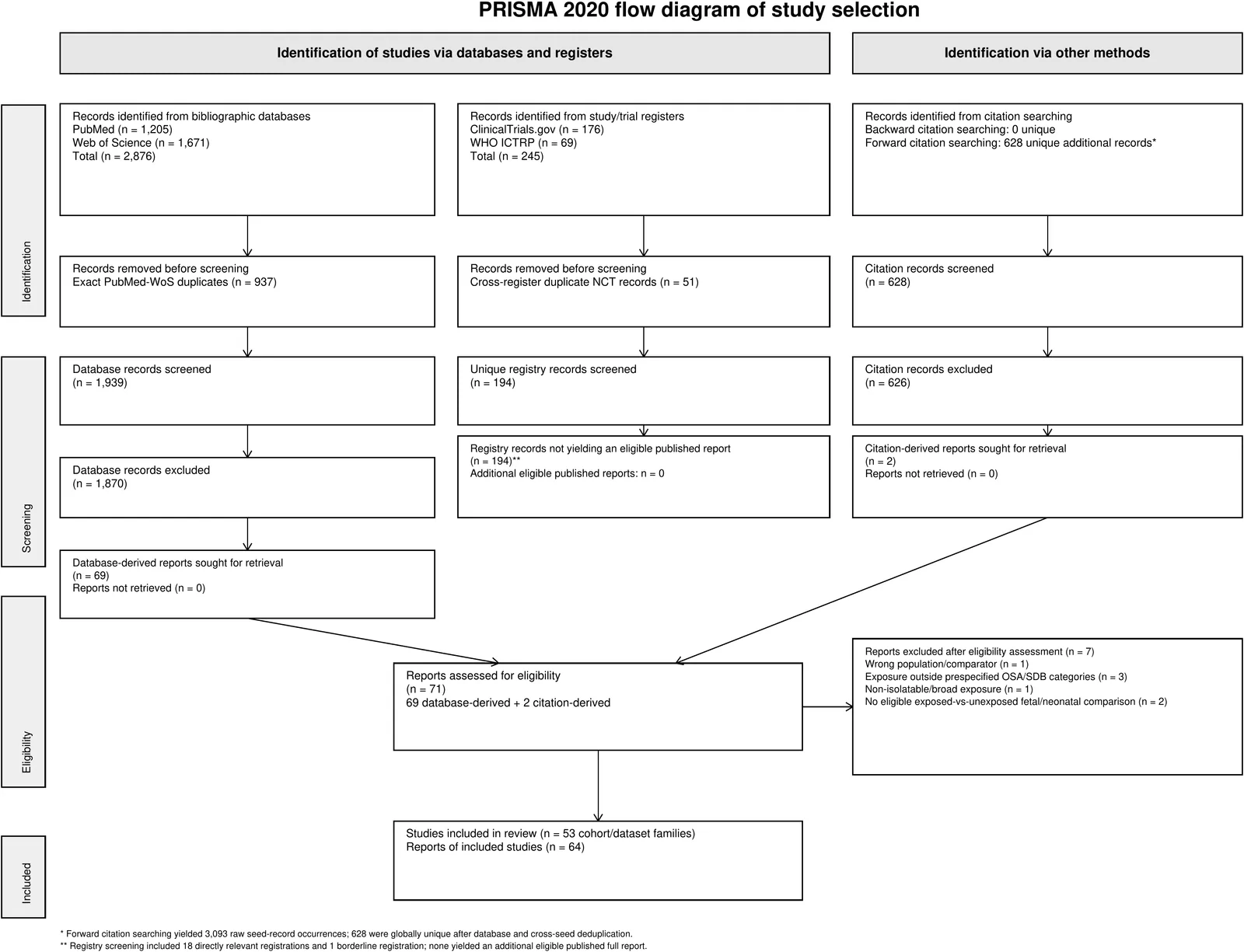
PRISMA 2020 flow diagram of study selection. Adapted from Page et al. (2021), licensed under CC BY 4.0.

### 2.4 Data extraction

A standardized extraction form was used by two investigators independently. For each publication, the revised extraction includes the unique cohort or dataset, recruitment period and setting, study design, total and exposed sample sizes, exposure ascertainment method, diagnostic or screening threshold, gestational timing, OSA/SDB severity, pre-existing versus pregnancy-onset symptoms where reported, PAP treatment status, singleton versus multiple gestation, population risk profile, outcome definitions and denominators, unadjusted and adjusted effect estimates, 95% confidence intervals, covariates in the preferred adjusted model, missing outcome data, and risk-of-bias considerations. The underlying study results were extracted rather than each paper’s narrative ‘main conclusion’. Discrepancies were resolved by discussion and consensus between the two reviewers, and no third-party adjudication was required. Publications from the same cohort were explicitly linked in a study-to-report map (**Supplementary Table 6**).

### 2.5 Risk of bias assessment

Two reviewers independently assessed risk of bias. For cohort and case–control studies, the Newcastle–Ottawa Scale (NOS) domains of selection, comparability, and outcome/exposure ascertainment were used as the core framework, but the summed score was not used as a standalone label for overall risk of bias. For included cross-sectional reports, an NOS-informed domain adaptation was used rather than assigning an unvalidated numerical NOS score. The assessment explicitly considered participant selection, exposure ascertainment and misclassification, confounding and covariate adjustment, outcome ascertainment, missing data, selective reporting or multiplicity, and exposure timing/severity. Overall concern was based on the domain profile rather than a numerical threshold. All 64 publication-level domain assessments were independently confirmed by the second reviewer, with no unresolved reviewer disagreements.

### 2.6 Outcomes

Primary outcome domains were birth anthropometrics (birth weight and length), FGR/IUGR, SGA, LGA, gestational age at delivery and preterm birth, Apgar scores at 1 and 5 minutes, and neonatal adiposity/body composition. Outcome definitions were retained as reported by each primary study; FGR, IUGR, SGA, low birth weight, and reduced growth velocity were not treated as interchangeable constructs. One-minute and five-minute Apgar findings were synthesized separately.

### 2.7 Data synthesis

Studies were grouped first by exposure ascertainment—objectively confirmed OSA/SDB, administrative-code-defined OSA, and symptom/snoring/questionnaire-defined SDB risk—and then by outcome. The revised synthesis does not classify studies solely as statistically significant or nonsignificant and does not use vote counting. For each outcome, the synthesis considers the number of participants and exposed participants, outcome definition, exposure timing and severity, unadjusted and adjusted effect estimates, 95% confidence intervals, covariates in the preferred adjusted model, and domain-level risk-of-bias concerns. Publications from the same cohort or dataset are linked and are not counted as independent evidence for the same outcome. Quantitative pooling was not undertaken in the final synthesis because clinically comparable subsets remained limited and heterogeneous with respect to exposure ascertainment, timing/severity, outcome definitions, adjustment sets, and overlapping report families; individual estimates are therefore summarized without a pooled effect.

## 3 Results

### 3.1 Characteristics of the included studies

The audit identified 64 eligible publications published from 1996 through 2026, mapped to 53 conservative cohort/dataset families (55 in the overlap-sensitivity count). Of the 64 publications, 22 used objective/physiologic assessment, 11 used administrative or clinically recorded OSA, and 31 used symptom-, snoring-, or questionnaire-defined SDB risk. The evidence base ranged from small prospective PSG, respiratory-polygraphy, oximetry, and WatchPAT cohorts to population-based administrative datasets containing hundreds of thousands or millions of deliveries. Because several publications arose from shared cohorts or overlapping administrative datasets, participant numbers were not summed across publications as though all reports were independent. Studies were conducted across North America, Europe, Asia, Australia/New Zealand, and Israel. Study-level characteristics, exposure definitions, eligible outcomes, and extracted effect estimates for all 64 publications are provided in **Supplementary Table 3**.

Exposure ascertainment remained heterogeneous and was therefore preserved as three distinct evidence strata: objectively confirmed OSA/SDB, administrative-code-defined OSA, and symptom/snoring/questionnaire-defined SDB risk. Exposure timing also varied from pre-pregnancy or pregnancy-onset symptoms to early-, mid-, and late-pregnancy objective testing. These distinctions are carried through all outcome-level syntheses.

Several publications arose from shared cohorts or report families and were linked before outcome-level synthesis. Confirmed examples include Hawkins et al. (2021) and Delgado et al. (2024) from the nuMoM2b SDB substudy; Telerant et al. (2018) and Brener et al. (2020) from the Tel Aviv non-obese WatchPAT cohort; Passarella et al. (2021) and Frappaolo et al. (2023) within the overlapping US NIS/HCUP family; and Felder et al. (2017, 2023) within overlapping California linked birth/hospital datasets. Köken et al. (2007), Sahin et al. (2008) and Tauman et al. (2011, 2012) were conservatively treated as shared families for the primary family count. **Supplementary Table 6** documents the report-family mapping and specifies which report contributes each outcome estimate to avoid double counting.

### 3.2 Risk of bias assessment

Risk-of-bias interpretation was revised to avoid equating a summed NOS score with a blanket overall judgment. Across the evidence base, recurrent concerns included misclassification of SDB exposure, selection into objective sleep testing, residual confounding by obesity and pregnancy comorbidities, inconsistent adjustment sets, differences in outcome definitions and ascertainment, small sample sizes in several objectively tested cohorts, administrative-code under-ascertainment of undiagnosed OSA, and incomplete consideration of exposure timing and severity. Domain-level judgments and supporting reasons are reported for each included publication, and outcome-level confidence is interpreted in light of these concerns. Publication-level domain judgments are provided in **Supplementary Table 2**, and outcome-level confidence considerations are summarized in **Supplementary Table 4**.

### 3.3 Birth anthropometrics

Twenty-four publications reported birth-anthropometric outcomes. Among objectively assessed studies, the direction of association was inconsistent. Telerant et al. (2018) reported a higher birth-weight percentile with mild late-pregnancy OSA (72% vs 57%, p<0.01) and more LGA births (28% vs 8%, p=0.04), whereas in the nuMoM2b substudy the unadjusted association between early-pregnancy SDB and LGA (RR 2.2, 95% CI 1.3-3.5) was attenuated after adjustment for maternal BMI (adjusted RR 1.0, 95% CI 0.6-1.8) (Hawkins et al., 2021). Alonso-Fernández et al. (2021) found similar birth weight in PSG-defined OSA and non-OSA groups (3.25 vs 3.21 kg, p=0.963), and Ghesquière et al. (2020) and Moro et al. (2026) likewise reported no clear OSA-group difference in fetal/neonatal growth measures. Redhead et al. (2026) reported that more frequent >3% oxygen desaturations were associated with higher birth weight (adjusted beta 0.75, 95% CI 0.20-1.29), indicating that objective respiratory metrics did not show a uniform association with lower birth size. Administrative-code studies more often suggested reduced birth size, although the magnitude was modest or outcome-specific. Bourjeily et al. (2020) reported a mean birth-weight difference of -48 g (95% CI -90 to -7) in pregnancies with coded OSA, and Chen et al. (2012) reported increased odds of low birth weight (adjusted OR 1.76, 95% CI 1.28-2.40). Additional studies reported heterogeneous associations of maternal snoring or OSA with birth size and fetal wellbeing (Ayrım et al., 2011; Franklin et al., 2000; Howe et al., 2015; Ko et al., 2013; Loube et al., 1996; Salameh et al., 2018; Sari et al., 2022; Yin et al., 2008). Symptom-defined studies were also heterogeneous: severe late-pregnancy snoring was associated with low birth weight in the Rhea cohort (adjusted RR 2.6, 95% CI 1.2-5.4) (Micheli et al., 2011), whereas Tauman et al. (2011, 2012), Sarberg et al. (2014), and Peltonen et al. (2023) did not identify a clear adverse birth-weight association. Overall, birth-anthropometric findings varied by exposure construct and adjustment, particularly for maternal adiposity, and did not support a consistent direction of effect.

### 3.4 Fetal growth restriction and small for gestational age status

Thirty publications contributed FGR, SGA, LGA, or related fetal-growth outcomes, and the re-audited findings showed signals in both restricted- and accelerated-growth directions. In objective studies, Fung et al. (2013) reported impaired fetal growth with maternal OSA (RR 2.67, 95% CI 1.25-5.70), although the BMI-adjusted OR was imprecise (5.3, 95% CI 0.93-30.34). Kneitel et al. (2018) reported increased odds of impaired fetal growth (adjusted OR 3.9, 95% CI 1.2-12.6) and third-trimester growth slowing (adjusted OR 3.6, 95% CI 1.4-9.4). In the Canadian 3D Birth Cohort substudy, PSG-defined SDB was associated with SGA across several AHI thresholds, including AHI ≥ 10 (adjusted OR 2.65, 95% CI 1.15-6.10), although the sampling strategy selected pregnancies with predicted fetal weight below the 75th centile and therefore limits generalizability (Pamidi et al., 2016). Conversely, Telerant et al. (2018) reported accelerated growth/LGA, while Hawkins et al. (2021) found no independent SGA/LGA association after BMI adjustment and Facco et al. (2014) found no association of early- or late-pregnancy SDB with SGA/LGA extremes.

Administrative-code findings were similarly nonuniform for growth phenotype. Chen et al. (2012) reported increased SGA (adjusted OR 1.34, 95% CI 1.09-1.66), whereas Bin et al. (2016) reported increased LGA (adjusted RR 1.27, 95% CI 1.04-1.55) but not SGA (adjusted RR 0.81, 95% CI 0.61-1.08), and Bourjeily et al. (2020) reported no clear SGA association (adjusted OR 0.97, 95% CI 0.82-1.15). Among symptom-defined studies, chronic snoring was associated with SGA in the Michigan cohort (adjusted OR 1.65, 95% CI 1.02-2.66) (O'Brien et al., 2013), pregnancy-onset snoring was associated with LGA in a large Chinese cohort (adjusted RR 1.71, 95% CI 1.31-2.24) (Ge et al., 2016), and prepregnancy snoring was associated with FGR in one cohort (adjusted OR 5.06, 95% CI 1.68-15.20) (White et al., 2022). In contrast, Tauman et al. (2012) found no association between maternal snoring and fetal growth. Thus, the growth evidence did not indicate a single restricted-growth phenotype attributable to maternal SDB.

### 3.5 Gestational age and preterm birth

Twenty-eight publications reported gestational-age or preterm-birth outcomes. Objective evidence for preterm birth was limited and inconsistent: Facco et al. (2012) reported increasing composite adverse-pregnancy-outcome rates across AHI categories (18.1%, 23.5%, and 38.5%; p=0.038), but a later high-risk objective cohort found no association of early- or late-pregnancy SDB with preterm birth <34 weeks (Facco et al., 2014). Pamidi et al. (2016) reported no clear AHI-stratum association with preterm birth (p=0.3), and Moro et al. (2026) and Sarberg et al. (2016) did not identify a clear objective-SDB association with gestational age/preterm delivery.

Administrative-code OSA showed the most recurrent preterm-birth signal, although several reports arose from overlapping datasets and coded exposure may under-ascertain undiagnosed disease. Reported adjusted estimates included OR 2.6 (95% CI 1.02-6.6) in Louis et al. (2010), OR 2.31 (95% CI 1.77-3.01) in Chen et al. (2012), OR 1.90 (95% CI 1.09-3.30) in Spence et al. (2017), and OR 1.48 (95% CI 1.29-1.69) in Bourjeily et al. (2020). In California linked data, coded sleep apnea was associated with birth before 37 weeks (OR 1.5, 95% CI 1.2-1.8) and more strongly with birth before 34 weeks (OR 2.2, 95% CI 1.5-3.1) (Felder et al., 2017). Symptom/questionnaire studies were heterogeneous but included positive associations for gasping (adjusted OR 1.8, 95% CI 1.1-3.2) (Bourjeily et al., 2010), Berlin Questionnaire high risk (overall preterm delivery adjusted OR 2.00, 95% CI 1.20-3.34; spontaneous preterm delivery adjusted OR 2.45, 95% CI 1.20-5.02) (Na-Rungsri et al., 2016), prepregnancy snoring (adjusted OR 2.75, 95% CI 1.10-6.92) (White et al., 2022), and a positive STOP screen (adjusted OR 2.8, 95% CI 1.4-5.8) (Seybold et al., 2022). Pregnancy-onset snoring in White et al. was not associated with preterm birth, and Robertson et al. (2022) did not identify an association with birth before 37 weeks. Differences in exposure timing, obesity, hypertensive disorders, and adjustment sets likely contributed to the variability. Other studies using symptom or screening measures also reported heterogeneous gestational-age and perinatal findings (Antony et al., 2014; Olivarez et al., 2011; Okun and O’Brien, 2018; Sharma et al., 2016; Smyka et al., 2022; Ugur et al., 2012).

### 3.6 Apgar scores and other neonatal outcomes

Twenty-three publications reported Apgar outcomes and ten reported NICU admission or other neonatal morbidity. Objective-study findings were mixed. In the very small PSG study by Sahin et al. (2008), only four women had OSA; their neonates had lower reported birth weight/Apgar scores and three of four were admitted to a newborn-care unit, making the estimate highly unstable. Bassan et al. (2016) found no difference in 5-minute Apgar (9.8 ± 0.6 vs 9.9 ± 0.3), Alonso-Fernández et al. (2021) found no 1- or 5-minute Apgar difference, and Ghesquière et al. (2020) reported no clear fetal/neonatal group difference. Redhead et al. (2026) reported increased odds of 5-minute Apgar <7 with objectively assessed OSA (adjusted OR 18.41, 95% CI 1.17-289.29), but the extremely wide confidence interval indicates substantial imprecision. In an obese objective-monitor cohort, NICU admission was more frequent with OSA (46.1% vs 17.8%, p=0.002), although the exposed group also had greater obesity and chronic hypertension (Louis et al., 2012). New-onset mid-pregnancy SDB in the nuMoM2b substudy was associated with a composite neonatal morbidity outcome (adjusted RR 1.43, 95% CI 1.05-1.94) (Delgado et al., 2024). Other physiologic or clinically defined cohorts also reported heterogeneous perinatal findings in populations characterized by maternal oxygen saturation, gestational diabetes, or hypertensive disorders (Moncadas et al., 2026; Orabona et al., 2024; Wilson et al., 2020).

Administrative-code studies identified selected neonatal associations but not a uniform Apgar effect (Louis et al., 2014). Bin et al. (2016) reported increased risk of 5-minute Apgar <7 (adjusted RR 1.60, 95% CI 1.07-2.38) and NICU/special-care admission (adjusted RR 1.26, 95% CI 1.11-1.44). In propensity-matched California data, Felder et al. (2023) reported increased 1-minute Apgar <7 (OR 1.6, 95% CI 1.3-2.0), whereas the 5-minute estimate was imprecise and included the null (OR 1.4, 95% CI 0.9-2.2); many crude neonatal associations attenuated after matching. Symptom/questionnaire evidence was also inconsistent: Tauman et al. (2011); Sarberg et al. (2014); Higgins et al. (2011), and Peltonen et al. (2023) found no clear adverse Apgar association, whereas a positive STOP screen predicted NICU admission (OR 2.5, 95% CI 1.1-5.7) (Seybold et al., 2022), and Komada et al. (2025) and Saha et al. (2026) reported lower Apgar findings without extractable SDB-specific effect sizes. The single birth/early-adiposity report from the Tel Aviv WatchPAT family found a smaller birth head circumference in the SDB group (p=0.004), but the follow-up sample was small (Brener et al., 2020).

### 3.7 Summary of findings

Across the 64 publications, no single fetal/neonatal outcome showed a consistent pattern across all three SDB ascertainment strata. The most recurrent association was preterm birth in administrative-code cohorts and in several symptom/questionnaire cohorts, but these findings are constrained by coding misclassification, residual confounding, heterogeneous symptom definitions, and overlapping datasets. Objective studies provided important signals for fetal-growth alteration and selected neonatal outcomes, yet results varied in direction and were frequently based on small or selected populations; several objective cohorts reported null findings. Growth findings included both FGR/SGA and LGA/higher-birth-weight phenotypes, arguing against a uniform growth-restriction interpretation. Apgar and neonatal-morbidity findings were sparse and, when positive, were sometimes markedly imprecise. Accordingly, the synthesis emphasizes effect magnitude and precision, exposure timing and severity, adjustment strategy, and cohort independence rather than counts of statistically significant reports.

### 3.8 Comparison by SDB assessment method

Descriptive comparisons by SDB ascertainment method are retained because objective testing, administrative coding, and symptom/questionnaire definitions measure different exposure constructs. Findings are therefore presented within, not across, these strata unless a clinically defensible comparison can be made.

Objective studies are interpreted in relation to testing method, AHI/RDI threshold, timing and severity; administrative studies are interpreted in light of diagnostic under-ascertainment and coding limitations; and symptom/snoring studies are interpreted as evidence about SDB symptoms or risk rather than diagnosed OSA. Pre-existing and pregnancy-onset symptoms are separated where the primary report permits this distinction.

No causal or mechanistic explanation is inferred from differences between exposure strata. Differences in sample size, statistical power, population risk, timing, confounder adjustment, outcome definitions, and exposure misclassification may all contribute to apparently different patterns across methods.

## 4 Discussion

This systematic review of 64 publications mapped to 53 conservative cohort/dataset families found no uniform fetal or neonatal risk pattern that could be attributed to maternal SDB across exposure definitions. Objectively confirmed OSA/SDB showed associations with fetal-growth alteration or selected neonatal outcomes in some cohorts, but several other objectively tested studies were null, and positive estimates were often imprecise or derived from selected high-risk populations. Administrative-code OSA showed the most recurrent association with preterm birth and selected neonatal outcomes, while symptom/snoring/questionnaire studies were heterogeneous and appeared sensitive to the specific symptom and its timing. These three evidence strata therefore should not be interpreted as interchangeable measures of one exposure.

The growth evidence particularly illustrates why a simple adverse-versus-null classification is misleading. Objective and symptom-based studies reported associations with FGR or SGA in some settings, whereas other studies reported LGA, higher birth-weight percentile, or higher birth weight. In nuMoM2b, an unadjusted early-SDB association with LGA was eliminated after BMI adjustment, highlighting maternal adiposity as a major confounder. Conversely, selected objective studies of fetal-growth trajectory retained associations after adjustment but had wide confidence intervals or restricted sampling frames. The available human evidence therefore does not support a single growth-restriction pathway as the characteristic fetal phenotype of maternal SDB.

Preterm birth was the outcome with the most recurrent positive estimates, particularly in administrative datasets. However, administrative coding identifies diagnosed disease rather than all physiologic OSA, provides limited information on gestational timing, severity, or treatment, and may be strongly correlated with obesity and other comorbidities. Several large administrative reports also arise from overlapping national or state datasets and cannot be counted as independent replications. Symptom-based preterm findings likewise varied by exposure definition: gasping, a positive Berlin/STOP screen, and prepregnancy snoring were associated with preterm birth in selected studies, whereas pregnancy-onset snoring and other symptom cohorts were null. This pattern suggests that exposure definition and timing materially influence the observed association.

Risk of bias is outcome- and estimand-dependent. Maternal obesity, age, smoking, parity, diabetes, hypertension, socioeconomic conditions, and multiple gestation may confound associations between SDB and fetal/neonatal outcomes. Hypertensive disorders and gestational diabetes may in some settings lie on the causal pathway rather than act solely as baseline confounders; therefore, models that adjust for every pregnancy complication are not automatically preferred. Small objective cohorts additionally face imprecision and selection into sleep testing, whereas administrative studies face diagnostic under-ascertainment and symptom studies face exposure misclassification. These limitations explain why a precise administrative association is not necessarily stronger causal evidence than a less precise objective estimate.

Prior systematic reviews and meta-analyses have synthesized maternal SDB in relation to perinatal and growth outcomes (Pamidi et al., 2014; Ding et al., 2014; Brown et al., 2018; Li et al., 2018; Liu et al., 2019; Sanapo et al., 2024). The present re-audit changes the interpretation in three ways: it expands and reconciles the primary evidence set, separates physiologically confirmed disease from coded diagnosis and symptom-defined risk, and maps overlapping publications to their underlying cohorts before interpreting outcome-level estimates. This approach reduces the risk that large report families or repeated publications are inadvertently treated as multiple independent confirmations.

Biological plausibility remains relevant context. Intermittent hypoxemia, oxidative stress, inflammation, sympathetic activation, altered placental perfusion, and metabolic dysregulation provide plausible pathways linking maternal SDB to fetal development. However, mechanistic plausibility cannot substitute for consistent human association evidence and does not establish that observed associations are causal.

This review does not evaluate the diagnostic accuracy, benefits, harms, or cost-effectiveness of universal SDB screening, and it does not synthesize controlled evidence of PAP treatment efficacy for fetal or neonatal outcomes. Accordingly, the observed associations—particularly those from administrative or questionnaire-defined exposures—do not by themselves justify a recommendation for universal screening or establish that PAP treatment reduces fetal/neonatal risk. Screening and treatment remain important clinical and research questions, but they require evidence designed specifically to address those questions.

Future studies should use clearly specified objective diagnostic criteria, assess exposure prospectively at defined gestational time points, report AHI/RDI severity and hypoxemic burden, distinguish pre-existing from pregnancy-onset SDB, and report treatment status. Standardized definitions for SGA, FGR, LGA, preterm birth, low Apgar, and neonatal morbidity are needed, together with prespecified confounder sets that distinguish baseline confounding from possible mediators. Adequately powered prospective cohorts and intervention studies are required to determine whether fetal/neonatal associations depend on SDB timing, severity, phenotype, or treatment.

In summary, the certainty of the fetal and neonatal evidence remains limited by heterogeneous exposure definitions, inconsistent confounder adjustment, imprecision in several objectively tested cohorts, exposure misclassification in questionnaire and administrative studies, outcome heterogeneity, and report overlap. The evidence supports a cautious outcome-specific interpretation: preterm birth has the most recurrent positive signal, whereas fetal-growth findings are bidirectional and Apgar/neonatal-morbidity findings are less consistent. These limitations are reflected in the Conclusions and preclude a single pooled causal interpretation of maternal SDB and fetal/neonatal outcome risk.

## 5 Conclusions

Across 64 eligible publications representing 53 conservative cohort/dataset families, current evidence is insufficient to establish a consistent association between objectively confirmed maternal OSA/SDB and the fetal or neonatal outcomes examined. Objective studies reported selected associations with fetal growth and neonatal outcomes, but null findings were also common and several estimates were imprecise. Administrative-code-defined OSA showed the most recurrent association with preterm birth and selected neonatal morbidity, while symptom/snoring/questionnaire-defined SDB risk produced heterogeneous findings that varied by exposure definition and timing.

Fetal-growth findings did not show a uniform direction, with both restricted-growth and LGA/higher-birth-weight associations reported. Apgar and other neonatal outcomes were inconsistently associated with SDB. Because of residual confounding, exposure misclassification, heterogeneous outcome definitions, small objective samples, and overlapping datasets, these observational findings should not be interpreted as establishing causality. The available evidence does not support a practice recommendation for universal SDB screening or a conclusion that PAP treatment improves fetal or neonatal outcomes, because those questions were not directly evaluated in this review.

Future research should prioritize adequately powered prospective objective exposure assessment across gestation, transparent reporting of severity and hypoxemic burden, treatment status, standardized fetal/neonatal outcome definitions, and prespecified confounder sets. Such studies are needed to determine whether specific SDB phenotypes, timing, or severity are associated with clinically meaningful fetal or neonatal risk and whether treatment modifies those outcomes.

## Data Availability

All data supporting the findings of this systematic review are contained within the article and its Supplementary Material. The study-level extraction, risk-of-bias assessments, complete search strategies, protocol-amendment table, and study-to-report map are provided in the **Supplementary Material**. No external repository is claimed for data generated by this review. Further inquiries can be directed to the corresponding author.
